# Combining nanoparticle albumin-bound paclitaxel with camrelizumab in advanced soft tissue sarcoma: activity, safety, and future perspectives

**DOI:** 10.3389/fphar.2024.1335054

**Published:** 2024-02-01

**Authors:** Zhichao Tian, Yushen Feng, Yang Yang, Xu Liu, Guoxin Qu, Yonghao Yang, Xin Wang, Jiaqiang Wang, Peng Zhang, Weitao Yao

**Affiliations:** ^1^ Department of Bone and Soft Tissue, The Affiliated Cancer Hospital of Zhengzhou University and Henan Cancer Hospital, Zhengzhou, China; ^2^ School of medicine, Case Western Reserve University, Cleveland, OH, United States; ^3^ Modern educational technology center, Henan University of Economics and Law, Zhengzhou, China; ^4^ Department of Immunotherapy, The Affiliated Cancer Hospital of Zhengzhou University and Henan Cancer Hospital, Zhengzhou, China

**Keywords:** nab-paclitaxel, camrelizumab, PD-1 inhibitor, sarcoma, immunochemotherapy

## Abstract

**Background:** It is still uncertain whether Nanoparticle albumin-bound paclitaxel (nab-paclitaxel) and programmed cell death protein 1 (PD-1) inhibitor have synergistic effects on metastatic soft tissue sarcomas (STSs). The purpose of this study was to evaluate the safety and activity of nab-paclitaxel plus camrelizumab (a PD-1 inhibitor) in patients with advanced STS who had previously failed chemotherapy.

**Methods:** In this single-center, open-label, single-arm phase II clinical trial, patients with advanced (unresectable or metastatic) STS who had previously failed chemotherapy received up to six cycles of nab-paclitaxel plus camrelizumab, whereas camrelizumab treatment was continued for up to 1 year. The median progression-free survival (PFS), objective response rate (ORR) and safety were collected and evaluated.

**Results:** This trial included 40 patients (28 men and 12 women). The overall ORR was 22.5%, and the median PFS was 1.65 months (95% confidence interval [CI], 1.3–2.0 months). Patients with epithelioid sarcoma demonstrated a longer PFS compared with those with other histological subtypes (2.3 months vs. 1.5 months, respectively); however, this difference was not significant. Patients who had received only one line of previous chemotherapy had a significantly longer PFS compared with those who had undergone two or more lines of previous chemotherapy (2.8 months vs. 1.3 months, respectively, *p* = 0.046). In terms of safety, the toxicity of this combination therapy is mild and no serious adverse events have occurred.

**Conclusion:** Nab-paclitaxel plus camrelizumab exhibited modest activity and mild toxicity in treating epithelioid sarcoma, angiosarcoma, and fibrosarcoma. The overall effectiveness of this treatment regimen for advanced STS is relatively low. Further research on combining nab-paclitaxel with effective drugs, including chemotherapy and targeted agents, for these specific STS subtypes is needed.

## 1 Introduction

Soft tissue sarcomas (STSs) are malignancies with a low incidence rate (approximately four per 100000 people) and high heterogeneity (>70 subtypes) (Yang et al., 2019; WHO Classification of Tumours, 2020; Buja et al., 2023). Moreover, approximately 50% of STS cases eventually progress to late stages. Traditionally, the main treatment method for advanced STS is chemotherapy, with first-line and second-line chemotherapy regimens including doxorubicin and docetaxel plus gemcitabine (Tian and Yao, 2023; von Mehren et al., 2020; George, 2019). However, the objective response rate (ORR) for this regimen is approximately 20%, and the median overall survival for patients with advanced STS is approximately 12 months (Tian and Yao, 2023). Therefore, effective treatment strategies are needed.

Nanoparticle albumin-bound paclitaxel (nab-paclitaxel) is a anticancer drug of taxane family (Yared and Tkaczuk, 2012; Kudlowitz and Muggia, 2014). It is a nano-sized paclitaxel, and has higher water solubility and bioavailability, lower toxicity, and improved antitumor efficacy compared with the two main taxanes (traditional paclitaxel and docetaxel) (Tian and Yao, 2022a; Mercatali et al., 2022). Nab-paclitaxel has been used to treat many types of cancer. In addition, recent reports have shown that it is effective in treating STSs (Tian et al., 2022a; Tian and Yao, 2022a).

Programmed cell death protein 1 (PD-1) inhibitors are the most widely used immunotherapy drugs in anticancer therapy, and they also have been used as novel antitumor therapies in the treatment and research of STS (Saerens et al., 2021). Despite recent evidence suggesting the low efficacy of PD-1 inhibitor monotherapy in STSs, there are promising reports of its effectiveness in some histological subtypes of sarcoma (Baldi et al., 2022; Kerrison et al., 2022). In addition, in order to improve the efficacy of PD-1 inhibitor, combination chemotherapy has been proven to be a promising method for treating malignancies (including STS) (Tian and Yao, 2022b).

Nab-paclitaxel plus PD-1 inhibitor has achieved promising results in the treatment of various types of cancer (Li et al., 2021; An et al., 2023; Sonoda et al., 2023; Yin et al., 2023; Zhang et al., 2023). However, clinical trials evaluating the efficacy and safety of this combination for STS treatment have not been reported. We have carry out a single-center, open-label, single-arm phase II clinical trial that used nab-paclitaxel plus camrelizumab (a PD-1 inhibitor) as a second-line treatment for metastatic or locally unresectable STS. We report the results of this trial here, and hoped to provide references for the treatment and clinical research for patients with STS.

## 2 Materials and methods

### 2.1 Patients

In this trial, the effects of nab-paclitaxel plus camrelizumab as a second or subsequent line of therapy for advanced STS were assessed. All patients received nab-paclitaxel plus camrelizumab between January 2022 and March 2023. The main eligibility criteria included: 1) age ≥18 years and <70 years, 2) histologically proven STS [include undifferentiated pleomorphic sarcoma (UPS), leiomyosarcoma, angiosarcoma, synovial sarcoma, clear cell sarcoma, epithelioid sarcoma, fibrosarcoma, malignant peripheral nerve sheath tumor (MPNST) and undifferentiated or poorly differentiated liposarcoma], 3) an Eastern Cooperative Oncology Group (ECOG) performance status of 0–1, 4) locally unresectable or multiple metastatic disease, 5) failure of previous chemotherapy, 6) acceptable hematological, renal and hepatic functions, 7) measurable lesions according to the response evaluation criteria in solid tumors (RECIST; version 1.1).

This study was approved by the Ethics Committee of the Henan Cancer Hospital and registered at ClinicalTrials.gov (NCT05189483). Written informed consent was obtained from all patients. This trial was performed in accordance with Good Clinical Practice guidelines and the Declaration of Helsinki.

### 2.2 Treatment protocol

All patients received 260 mg/m^2^ of nab-paclitaxel (Hengrui Pharmaceutical, Lianyungang, China), via a 30-min intravenous infusion on day 1, and repeated every 3 weeks for up to six cycles or until the occurrence of progressive disease (PD) or unacceptable adverse events (AEs). All patients received 200 mg of camrelizumab (Hengrui Pharmaceutical, Lianyungang, China) via a 30-min intravenous infusion on day 1, and repeated every 3 weeks for up to 1 year unless there was PD or unacceptable AEs. Treatment could be delayed for a maximum of 2 weeks in the case of grade 3 or 4 AEs.

### 2.3 Evaluations

Patient demographics and characteristics were recorded. The RECIST (version 1.1) was used to assessed Activity. During the first 16 cycles, tumor imaging assessments were conducted every two cycles or immediately after obtaining a clear evidence of PD; Afterwards, tumor imaging assessments will be conducted every four cycles. Safety was evaluated according to the National Cancer Institute Common Terminology Criteria for Adverse Events (version 4.0). The main indicators included AEs and immune-related AEs (irAEs). The safe follow-up period for the subjects starts from the last dose and follows up every 30 days until 90 days after the last dose.

### 2.4 Statistical analysis

Perform all statistical analysis using SPSS (version 21.0; SPSS Inc., Chicago, Illinois, United States of America). The corresponding figures were drawn using Graph Prism 5.0 (GraphPad Software, San Diego, CA, United States of America). Two-tailed tests were performed at a significance level of α = 0.05, with *p* < 0.05 indicative of statistical significance. Subgroup comparisons of count date were performed using the chi-square test or Fisher’s exact test. The relationship between the variables and survival was assessed using Kaplan-Meier curves and the log-rank test’s subgroup differences in survival were assessed. The follow-up period was extended to 30 September 2023.

## 3 Results

### 3.1 Patient characteristics

From January 2022 to March 2023, a total of 40 patients with unresectable or metastatic STS were enrolled in this study, with 28 men and 12 women. The average age of the patients was 49.28 ± 14.17 years (Table 1). All patients had an ECOG performance status of 0 or 1. The histological subtypes included UPS (n = 10), epithelioid sarcoma (n = 8), fibrosarcoma (n = 7), angiosarcoma (n = 5), myxofibrosarcoma (n = 3), MPNST (n = 2), leiomyosarcoma (n = 2), synovial sarcoma (n = 2), and differentiated liposarcoma (n = 1). The primary tumor sites were predominantly the limbs, followed by the trunk. Most patients experienced lung metastases, with a small number (12.5%) of them being locally unresectable. All patients had previously received at least one line of chemotherapy (Table 1).

**TABLE 1:** Clinical characteristics of patients in this study.

Characteristics	No. (%) (n = 40)
Gender	
Female	12 (30.0%)
Male	28 (70.0%)
Age	49.28 ± 14.17
ECOG PS	
0	20 (50.0%)
1	20 (50.0%)
Histology	
UPS	10 (25.0%)
Epithelioid sarcoma	8 (20.0%)
Fibrosarcoma	7 (17.5%)
Angiosarcoma	5 (12.5%)
Myxofibrosarcoma	3 (7.5%)
MPNST	2 (5%)
Leiomyosarcoma	2 (5%)
Synovial sarcoma	2 (5%)
Dedifferentiated liposarcoma	1 (2.5%)
Primary site	
Extremities	26 (65.0%)
Trunk	13 (32.5%)
Head	1 (2.5%)
Stage	
IV	35 (87.5%)
Locally unresectable	5 (12.5%)
Metastatic site	
lungs	32 (80.0%)
other	3 (7.5%)
Lines of previous systemic therapy	
1	11 (27.5%)
2	15 (37.5%)
3	14 (35.5%)

Abbreviations: ECOG PS, eastern cooperative oncology group performance status; MPNST, malignant peripheral nerve sheath tumor; UPS, undifferentiated pleomorphic sarcoma.

### 3.2 Activity of treatment

**FIGURE 1:**
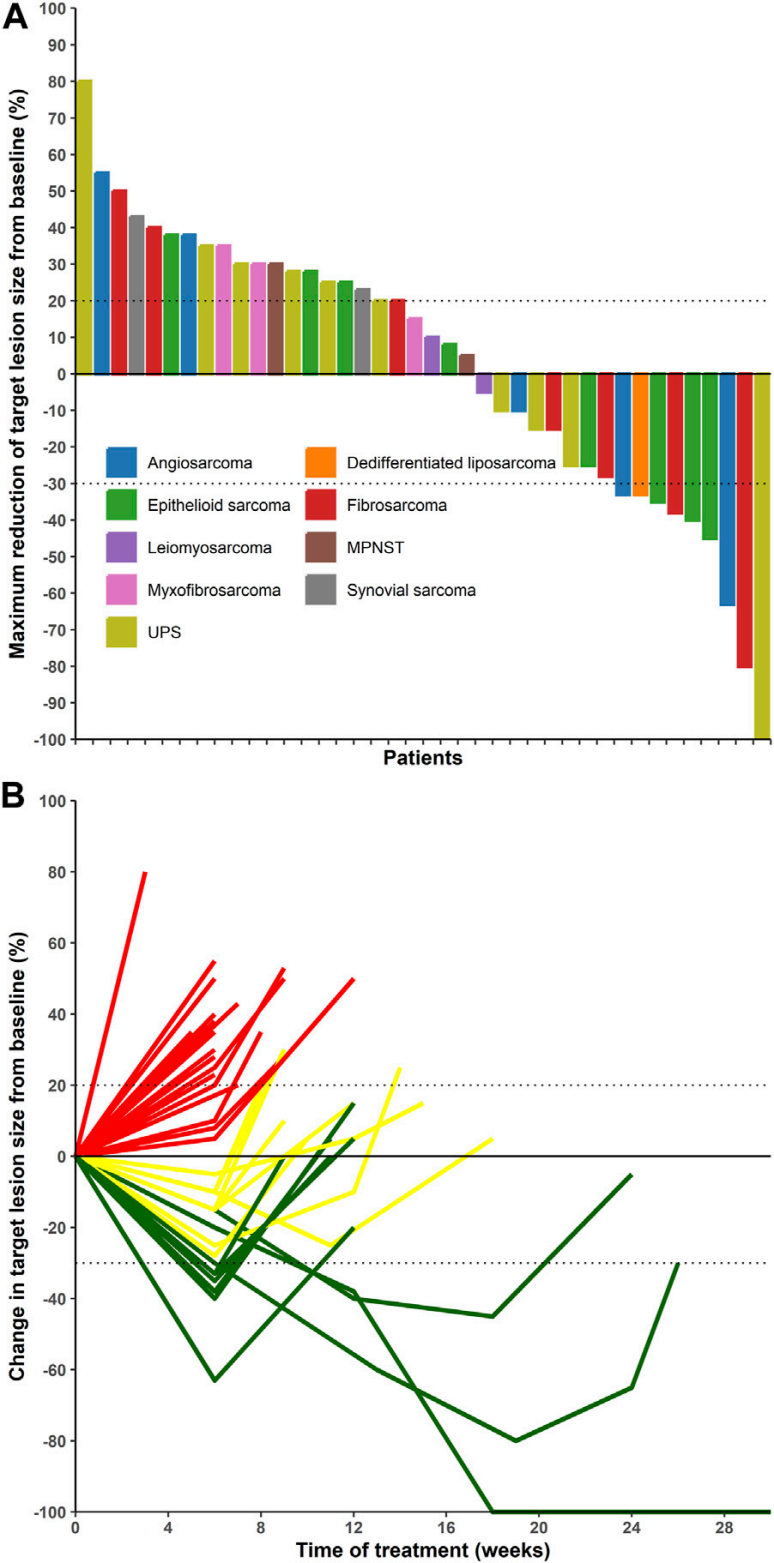
**(A)** Waterfall plot illustrating the maximum reduction in target lesion size from baseline, evaluated according to the response evaluation criteria for solid tumors (version 1.1). **(B)** Line plot showing the duration of response of target lesions from baseline. Abbreviations: MPNST, malignant peripheral nerve sheath tumor; UPS, undifferentiated pleomorphic sarcoma.

Among the 40 patients, 1 case of CR (UPS) and 8 cases of PR (3 epithelioid sarcomas, two fibrosarcomas, two angiosarcomas, and one dedifferentiated liposarcoma) were identified (Table 2; Figure 1). The ORR, DCR, median PFS, and 4-month PFS rates were 22.5%, 50%, 1.65 months, and 7.5%, respectively (Table 3 and Figure 1).

**TABLE 2:** Efficacy of various histological subtypes.

Histology	Number of patients			
	CR	PR	SD	PD
UPS (n = 10)	1	0	3	6
Epithelioid sarcoma (n = 8)	0	3	2	3
Fibrosarcoma (n = 7)	0	2	2	3
Angiosarcoma (n = 5)	0	2	1	2
Myxofibrosarcoma (n = 3)	0	0	1	2
MPNST (n = 2)	0	0	1	1
Leiomyosarcoma (n = 2)	0	0	1	1
Synovial sarcoma (n = 1)	0	0	0	2
Dedifferentiated liposarcoma (n = 1)	0	1	0	0
Total	1	8	11	20

Abbreviations: CR, complete response; PR, partial response; SD, stable disease; PD, progressive disease; MPNST, malignant peripheral nerve sheath tumor; UPS, undifferentiated pleomorphic sarcoma.

**TABLE 3:** Efficacy of all patients to the treatment.

Characteristics	Data
ORR (%)	22.50 (95% CI: 10.84–38.45)
DCR (%)	50.00 (95% CI: 33.80–66.20)
Median-PFS (months)	1.65 (95%CI: 1.30–2.00)
4-month PFS rate (%)	7.50 (95%CI: 1.94–18.24)
6-month PFS rate (%)	2.50 (95%CI: 0.20–11.27)

Abbreviations: ORR, objective response rate; DCR, disease control rate; PFS, progression-free survival; CI, confidence interval.

**FIGURE 2:**
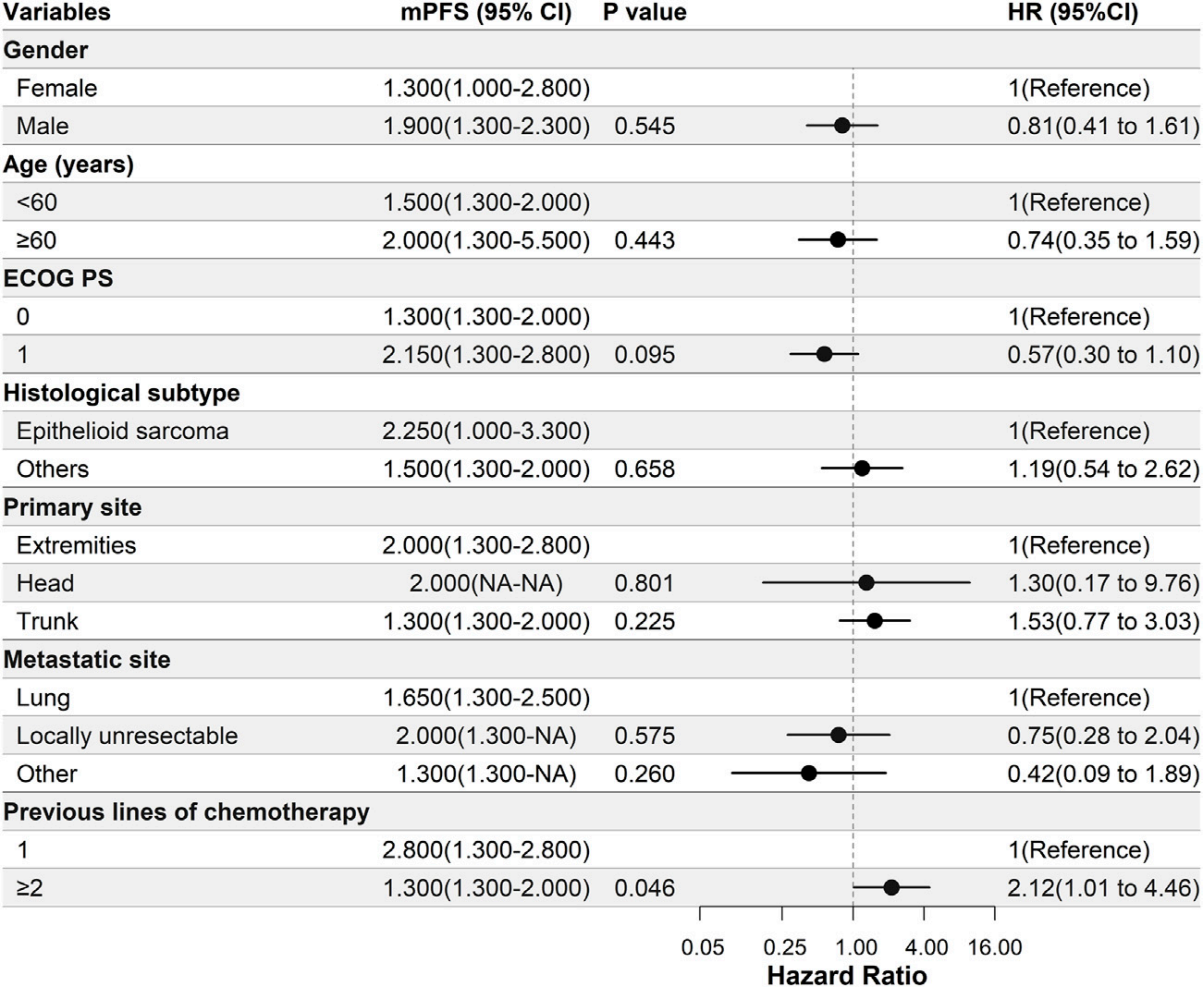
Univariate Cox regression analysis of the relationship between clinical parameters and progression-free survival (PFS). In this study, patients with epithelioid sarcoma had a longer PFS than those with other histological subtypes; however, there was no significant difference. Patients who underwent one line of previous chemotherapy had a significantly longer PFS compared with those who had undergone two or more lines of previous chemotherapy. Abbreviations: ECOG PS, Eastern Cooperative Oncology Group performance status; mPFS, median progression-free survival.

Patients with epithelioid sarcoma had a longer PFS than those with other histological subtypes (2.3 months vs. 1.5 months, respectively); however, this difference was not significant (Figure 2). Patients who had received only one line of previous chemotherapy had a significantly longer PFS compared with those who had undergone two or more lines of previous chemotherapy (2.8 months vs. 1.3 months, respectively, *p* = 0.046) (Figure 2).

### 3.3 Toxicity and safety

In general, nab-paclitaxel plus camdelizumab was well-tolerated (Table 4). Most AEs were associated with nab-paclitaxel treatment. The most common grade 1–2 AEs were alopecia (89.3%, 25/28), lymphocytopenia (75.0%, 30/40), leukopenia (55.0%, 22/40), anemia (32.5%, 13/40), and nausea (22.5%, 9/40). The most common grade 3 AEs were lymphocytopenia (22.5%; 9/40) and leucopenia (15.0%, 6/40). No grade 4 AEs were observed. IrAEs were mild and were of only two types: hyperthyroidism (15%, 6/40) and rash (5.0%, 2/40). No patient needed to reduce the dosage of nab-paclitaxel or PD-1 inhibitor due to AEs, and there were no treatment-related deaths.

**TABLE 4:** Adverse events.

Adverse events	All grades	Grade 3–4
Alopecia	90.0% (36/40)	
Lymphocytopenia	75.0% (30/40)	22.5% (9/40)
Leucopenia	55.0% (22/40)	15.0% (6/40)
Anemia	32.5% (13/40)	2.5% (1/40)
Nausea	22.5% (9/40)	
Numbness of limbs	20.0% (8/40)	2.5% (1/40)
Pain	20.0% (8/40)	2.5% (1/40)
Thrombocytopenia	17.5% (7/40)	2.5% (1/40)
Fatigue	17.5% (7/40)	
Transaminase increase	17.5% (7/40)	
Anorexia	15.0% (6/40)	
Hypothyroidism	12.5% (5/40)	2.5% (1/40)
Diarrhea	12.5% (5/40)	
Fever	7.5% (3/40)	
Rash	5.0% (2/40)	

## 4 Discussion

The study aimed to assess the activity and safety of nab-paclitaxel in combination with a PD-1 inhibitor, camrelizumab, as a second or subsequent line of therapy for advanced STS. Our results revealed noteworthy findings regarding this treatment approach.

Evidence suggests that chemotherapy can enhance the anti-tumor effects of PD-1 inhibitor by reducing the number of tumor cells, promoting immunogenic tumor cell death, consuming immunosuppressive cells, increasing the number and activity of anti-tumor immune effector cells, and enhancing the secretion of cytokines that promote immune cell proliferation (Principe et al., 2022; Zhu et al., 2022). Nab-paclitaxel is an immunogenic cell death inducer that has been shown to enhance the efficacy of PD-1 inhibitors by regulating various immune functions (Li et al., 2021; Yoneshima et al., 2021). Currently, chemotherapy combined with PD-1 inhibitor has been approved for the treatment of gastroesophageal, lung and breast cancers (Tian and Yao, 2022b; Principe et al., 2022).

In this study, although an ORR of 22.5%, comparable to that achieved with doxorubicin plus PD-1 inhibitors, was achieved (Pollack et al., 2020; Livingston et al., 2021; Tian et al., 2022b), the median PFS was only 1.65 months. This indicates that the treatment only elicited therapeutic effects for a brief initial period, and that there is no synergistic effect. Notably, a CR was observed in a patient with UPS. This response may be due to the well-documented sensitivity of UPS to PD-1 inhibitors, as indicated in previous studies (Moreno Tellez et al., 2022). The efficacy in the other histological subtypes can be attributed to nab-paclitaxel. Although the overall treatment effect is not satisfactory, this regimen exhibited therapeutic effects against epithelioid sarcoma, angiosarcoma, and fibrosarcoma, consistent with previous studies (Tian et al., 2022a). Owing to the observed short PFS for these STS histological subtypes, the use of nab-paclitaxel alone or in combination with a PD-1 inhibitor is not recommended for advanced STS treatment. Instead, consideration should be given to exploring combinations involving nab-paclitaxel with other effective drugs, such as chemotherapeutic and targeted drugs, for these subtypes.

In terms of safety, our findings revealed a relatively low incidence of AE, with rare occurrences of grade 3–4 AEs. This indicates that the combination of nab-paclitaxel and a PD-1 inhibitor is safe, consistent with previous studies (Hao et al., 2023), and notably better than first-line chemotherapy for STS (Gronchi et al., 2017; Seddon et al., 2017). This safety profile can greatly enhance patient satisfaction and quality of life during treatment. Given the lower incidence of AEs, considering the addition of other drugs, such as targeted drugs or chemotherapy drugs, to the protocol used in this study may improve treatment effectiveness.

Our study has certain limitations, including the absence of a control group, a relatively small number of patients, and a short follow-up period. Despite these limitations, this study demonstrated the limited efficacy of nab-paclitaxel plus camrelizumab in treating STSs. Non-etheless, nab-paclitaxel shows promise in treating epithelioid sarcoma, angiosarcoma, and fibrosarcoma. Therefore, further research investigating the use of nab-paclitaxel in combination with other effective drugs, such as chemotherapy and targeted drugs, for these specific subtypes of STSs is warranted.

## 5 Conclusion

Nab-paclitaxel plus camrelizumab exhibited modest activity and mild toxicity in treating epithelioid sarcoma, angiosarcoma, and fibrosarcoma. The overall effectiveness of this treatment regimen for advanced STS is relatively low. Further research on combining nab-paclitaxel with effective drugs, including chemotherapy and targeted agents, for these STS subtypes is needed.

## Figures and Tables

**Table:** 

**PD-1**	programmed cell death protein 1
**STS**	soft tissue sarcoma
**UPS**	undifferentiated pleomorphic sarcoma
**MPNST**	malignant peripheral nerve sheath tumor
**ECOG**	Eastern Cooperative Oncology Group
**RECIST**	response evaluation criteria in solid tumors
**AEs**	adverse events

## Data Availability

The original contributions presented in the study are included in the article/Supplementary material, further inquiries can be directed to the corresponding author.
